# Health funders’ dissemination and implementation practices: results from a survey of the Ensuring Value in Research (EViR) Funders’ Forum

**DOI:** 10.1186/s43058-022-00273-7

**Published:** 2022-03-29

**Authors:** Barbara van der Linden, Kelly M. Dunham, Joanna Siegel, Emily Lazowick, Michael Bowdery, Tara Lamont, Alison Ford

**Affiliations:** 1 ZonMw, P O Box 93245, 2509 AE The Hague, Netherlands; 2 Patient-Centered Outcomes Research Institute (PCORI), 1828 L St NW, Washington, DC, 20036 USA; 3 Health and Care Research Wales, Crown Buildings, Cathays Park, Cardiff, CF10 3NQ Wales, UK; 4 NIHR, University of Southampton, Southampton, UK

**Keywords:** Health research funders, Funding agencies, Dissemination and implementation practices, Knowledge exchange

## Abstract

**Background:**

A significant gap persists between evidence from research and its use in practice. Research funders, important actors in the health research system, can help reduce this gap by initiating dissemination and implementation (D&I) activities. The specific types of D&I activities funders currently lead have not been explored thoroughly. The Ensuring Value in Research (EViR) Funders’ Forum—an international collaboration of health-related research funders—was established in 2017 to address research waste issues and increase the value of research. The Forum surveyed funders to learn about their D&I practices and challenges.

**Methods:**

We distributed a five-item exploratory survey to participating funders in August 2018. The results informed the development of a survey instrument, distributed in June 2019. The survey instrument contained 15 items prompting respondents to categorize and describe their level of effort in six practice areas: release of findings, dissemination, knowledge exchange/partnering, implementation, building capacity, and implementation research. In addition, funders were asked to describe examples of their practices in detail. Thirty-one funders completed the survey instrument, a 58% response rate.

**Results:**

Most funders regard D&I as a high priority, but funders vary in levels of activity per practice area. Over half of respondents reported that they have at least some activity in all D&I practice areas surveyed, with the exception of implementation research. The vast majority indicated some or significant activity in release of findings (97%) and dissemination (87%). Nearly one-fifth of funders (19%) indicated that implementation is outside their remit, and 26% indicated that implementation research is outside their remit. Survey respondents shared a broad range of examples of activities in each practice area. Lack of evidence for successful approaches and measuring impact were named frequently as challenges and as potential areas for collaboration.

**Conclusions:**

Although models of dissemination and implementation vary across organizations, the majority of funders indicated that D&I of research findings is a priority. Funders indicated a need for evidence on effectiveness of various approaches to D&I. Increased collaboration between funders, including sharing good practices, will increase our collective learning and knowledge development.

**Supplementary Information:**

The online version contains supplementary material available at 10.1186/s43058-022-00273-7.

Contributions to the literature

- This manuscript describes international health research funders’ support for dissemination and implementation activities.
- The manuscript provides a practical framework of dissemination and implementation activities that is specific to health research funders.
- The survey findings indicate where opportunities remain for health research funders to promote uptake of evidence as well as highlighting current trends and progress to date.
- This information is not available elsewhere and can help inform funding approaches and practice among health research funders internationally.

## Background

The significant gap between evidence from research and its use in policy and practice has long been recognized [1]. Successful implementation of evidence generally occurs only after active effort and involves actors across the health research ecosystem, including researchers, health professionals, policy makers, the public, and research funders [2].

In recent years, a number of studies have explored funders’ roles in dissemination and integrating evidence into practice. Some of these have examined the activities of individual funders [3–5], others have examined the range of funder activities by country [6], and several studies have examined trends in funders’ dissemination and implementation initiatives internationally [7–9]. A recent study by McLean et al. [10], for example, reported on funders’ roles using data from 2012 to 2013. A majority of funders in this study indicated that knowledge transfer was an important priority, but most lacked clear investments in staffing or earmarked resources for knowledge translation. Funders also indicated a preference for “push” activities, designed to move research into the hands of appropriate end users, and “linkage and exchange” activities establishing partnerships between researchers and end users, rather than “pull” activities designed simply to facilitate user access to results.

While these studies have provided insight into funders’ interests and priorities, specific details of the range of funders’ activities—including the types of dissemination and implementation activities currently in use—have not been well described. The establishment of the Ensuring Value in Research (EViR) Funders’ Forum in 2017 [11] provided a unique opportunity to update knowledge on research funders’ efforts supporting the uptake of research findings into practice. The Forum was initiated to promote exchange and collaboration among research funders on the broad issue of reducing waste and increasing value in research and is open to health research funders, organizations that represent funders, and organizations that set health funding-related policy [12]. Individuals from 53 organizations have participated in Forum activities to date [13]. Funders agreed on guiding principles to address these issues, including one that focuses on the importance of supporting the use of findings from research [14]. Specifically, this principle states:

*Research knowledge that can lead to benefit should be effectively disseminated to end users. Where appropriate, the usage of new knowledge should be supported and facilitated.*

To characterize current dissemination and implementation (D&I) practices and identify opportunities to improve collaborative efforts, we established a working group, which included representatives from the Netherlands Organisation for Health Research and Development (ZonMw), the National Institute for Health Research (NIHR), Health and Care Research Wales, and the Patient-Centered Outcomes Research Institute (PCORI). This paper reports on the results of the working group’s 2019 survey of health research funders, describing current practices and further directions for funders’ work in this area.

## Methods

The Secretariat of the EViR Funders’ Forum maintains a mailing list of organizations who have selected to participate in the Forum activities. The list includes health-related research funders and organizations who set funding policy. Each organization has a primary point of contact, who is a senior leader representative from their organizations with cross-organizational reach and responsibilities.

In August 2018, we distributed a preliminary five-item, open-ended exploratory survey, in English (see Additional file 1) via email to the 32 funders on the EViR mailing list as of July 2018 to inform future efforts. Two funders were from the Australia-Pacific region, 22 were from Europe, and eight were from North America. The exploratory survey asked recipients to characterize current D&I practices, challenges associated with conducting D&I activities, and opportunities for collaboration. Fourteen funders completed the survey, a 44% response rate. The working group reviewed the content of the survey responses and identified broad themes and categories of D&I-related practices and challenges. The results were discussed at the November 2018 EViR Funders’ Forum meeting and in a follow-up webinar conducted to determine the implications of the responses and considerations for a structured survey. During these discussions, participants noted the importance of establishing definitions to clearly distinguish among dissemination, implementation, and other D&I-related practices, and endorsed circulating a structured survey to capture the full spectrum of funders’ D&I practices and challenges.

### Survey instrument

To develop the survey instrument, the working group drew on existing literature [3, 15–20] to create a practical framework that categorizes and describes D&I activities in six practice areas. We designed this framework to allow us to capture the range and diversity of funders’ activity in dissemination and implementation consistently across funders, and in ways more in line with the literature. Beginning with sources referenced in a 2015 review of the literature on D&I [17], we identified definitions of dissemination, implementation, and related terms; definitions of each category generally were similar across sources. The working group agreed on specific wording to be used in the framework. Then, based on input from the exploratory survey, the working group added related categories to capture activities that some funders described. For example, *Building Capacity/Infrastructure* describes investments some funders pursue—both in people and in structures—to lay needed groundwork for dissemination and implementation of evidence. Further, a number of funders had referenced their efforts in *Knowledge Exchange and Partnering*, to bring together stakeholders to share, respond to, and act upon research findings. In addition, we included the category *Implementation Research* as some funders described activities and investment in research on models and effectiveness of D&I efforts or initiatives.

The framework (Table 1) includes initial activities funders may take to promote the availability and access to findings of research they support (*Release of Findings*—defined as diffusion, passive activities, and supportive policies to make research findings available and accessible to the general public and other audiences), to those designed to promote active *Implementation*, defined consistent with the literature to include “active and planned efforts to use or integrate research findings within a setting.”

**Table 1:** Framework of practice areas: definitions and examples

Definition	Examples of activities in each D&I practice area
**Release of findings:** Diffusion, passive activities, and supportive policies to make research findings available and accessible to the general public and other audiences	• Publication/presentation of findings: funder requires or financially supports awardees to release findings in academic journals or at academic conferences • Open access: funder requires publication deposit in open access repositories; requires or provides funds for open access publication • Direct publication by the funder • Funder supports write-up (translation) of findings into lay language for the general public • Funder establishes information hub or repository for evidence to support its dissemination • Funder releases findings and information about funded projects in press briefings, media exchanges, untargeted mass mailings, untargeted presentations (e.g., meetings, webinars), leaflets, newsletters, blogs, web pages, and/or on social media
**Dissemination:** An active approach of spreading research findings to the target audience via determined channels using planned strategies [15]	• Prepare targeted summaries, briefings, or other products to disseminate evidence to stakeholders/targeted audiences • Educational sessions with patients, healthcare professionals, and/or policymakers • Develop or fund development of training modules that incorporate research findings for clinicians or other targeted audience • Develop or support development of clinical guidelines or other tools to increase knowledge or awareness of evidence in a targeted audience • Require or encourage research awardees to develop dissemination plans and/or conduct dissemination activities as part of research awards • Support awardees to conduct dissemination through separate funding schemes • Support or hold conferences, workshops, or other events to disseminate findings to targeted audiences • Funder undertakes other direct dissemination activities (directly reaches or works with stakeholders to actively reach targeted audiences).
**Knowledge exchange/partnering:** Actively bringing stakeholders together to share, respond to, and act upon research findings	• Stimulating partnerships between researchers and knowledge-users/local organizations to support the integration of research evidence into practice • Funder holds or supports knowledge exchange meetings/forums for researchers and decision makers • Knowledge translation, exchange, and/or mobilization funding schemes
**Implementation:** Active and planned efforts to use or integrate research findings within a setting	• Funding schemes to support awardees (research or other) to undertake implementation projects (e.g., clinical guideline implementation projects) • Require or encourage research awardees to develop implementation plans and/or conduct implementation activities as part of research awards • Direct support for implementation at implementation sites (funder hires or supports hiring an implementation expert(s) to implement intervention at new site) • Prizes to incentivize or recognize implementation projects
**Building capacity/infrastructure:** Investing in people and structures that enable/lay the groundwork for release of findings, dissemination, knowledge exchange/partnering, and/or implementation of research evidence	• Specific awards to support building capacity for dissemination or implementation activities • Funding to develop dissemination and implementation expertise (e.g., fellows) • Investment in networks, frameworks, tools, materials, etc. that support release of findings, dissemination, knowledge exchange/partnering, and/or implementation of research evidence
**Implementation research:** Investment in and/or carrying out research on determinants/models/working methods and effectiveness of dissemination and implementation efforts or initiatives	• Funder provides support for research having the primary aim of determining whether a dissemination or implementation strategy is effective • Funder provides support for research having the primary aim of comparing the effectiveness of proven dissemination or implementation strategies

The survey instrument contained 15 open- and closed-ended questions. In the survey, respondents were asked to categorize their level of effort and describe their activity in the six D&I practice areas: release of findings, dissemination, knowledge exchange/partnering, implementation, building capacity/infrastructure, and implementation research. To provide points of reference for respondents and promote comparability, the working group provided definitions of the practice areas as well as examples of activities in each area (Table 1). The survey also included questions about funding and staffing for D&I activities, challenges associated with conducting D&I activities, and opportunities for collaboration with other funders. Additional file 1 contains the survey instrument.

### Survey distribution

The survey was distributed electronically via SurveyGizmo to the representatives of the 53 health-related research funders on the EViR mailing list in June 2019. An additional file shows a list of all funders that received the exploratory survey and the survey instrument and characteristics of these (see Additional file 1). Five funders were from the Australia-Pacific region, 40 were from Europe, and eight were from North America. The survey was conducted in English and non-respondents were contacted by email once. Recipients were asked to work with colleagues in the organization, as appropriate, to complete the survey. Each recipient submitted only one response on behalf of the organization.

### Data analysis

We calculated frequency distributions for all closed-ended items. The working group reviewed the open-ended responses to assess alignment with framework categories. When respondents’ categorizations of activities in open-ended items did not clearly align with the provided definitions, the working group contacted the respondents by email for additional clarification. If the respondent did not reply to our first email inquiry, then we sent a second email one week later. We contacted 17 of the 31 respondents; 12 funders replied, and changes in categorizations were made for nine funders with their permission based on the clarifying information they provided. We did not modify the original responses among the five respondents that did not reply to our email inquiry.

This study was determined to be exempt from oversight by the Advarra institutional review board. However, participants agreed to remove organizational identifiers for specific examples provided in this manuscript.

## Results

### Response rate

Thirty-one funders from 12 countries completed the survey instrument, a 58% response rate (Table 2). Among the group of respondents, 58% were public or government-funded organizations, and 42% were philanthropic funders. In comparison, non-respondents included a lower proportion of public funders (41%) and a greater proportion of philanthropic organizations (59%). Among the respondents, 52% were from Europe (other than UK), 26% from the UK, 13% from North America, and 10% from the Australia-Pacific region. Survey recipients were asked to provide their organization’s annual budget. Among the 31 survey respondents, 52% (*N*=16) can be categorized as small funders with an annual budget of under €100M, 29% (*N*=9) as medium sized (annual budget between €100M and €500M), and 16% (*N*=5) as large funders with an annual budget above €500M. One respondent did not respond to this question.

**Table 2:** Survey respondents (*N*=31)

	Organization	Funder location (country)	Public vs. philanthropic
1.	Alzheimer Nederland	Netherlands	Philanthropic
2.	Aidsfonds	Netherlands	Philanthropic
3.	Australian Government Department of Health, Health and Medical Research Office	Australia	Public
4.	Brain Foundation Netherlands (Hersenstichting)	Netherlands	Philanthropic
5.	Canadian Institutes of Health Research	Canada	Public
6.	Diabetes Fonds	Netherlands	Philanthropic
7.	Dutch Heart Foundation (Hartstichting)	Netherlands	Philanthropic
8.	Dutch Kidney Foundation (Nierstichting Nederland)	Netherlands	Philanthropic
9.	Forte: Swedish Research Council for Health, Working Life and Welfare	Sweden	Public
10.	German Federal Ministry of Education and Research, DLR Project Management Agency	Germany	Public
11.	Health and Care Research Wales	UK	Public
12.	Health and Social Care in Northern Ireland, Public Health Agency	UK	Public
13.	Health Research Board Ireland	Ireland	Public
14.	Health Research Council of New Zealand	New Zealand	Public
15.	Marie Curie	UK	Philanthropic
16.	Medical Research Council	UK	Public
17.	MIND	Netherlands	Philanthropic
18.	Ministry of Health, Italy	Italy	Public
19.	National Health and Medical Research Council	Australia	Public
20.	National Institute for Health Research	UK	Public
21.	National Science Centre	Poland	Public
22.	Patient-Centered Outcomes Research Institute	USA	Public
23.	Princess Beatrix Muscle Fund (Prinses Beatrix Spierfonds)	Netherlands	Philanthropic
24.	Research Council of Norway	Norway	Public
25.	Scar Free Foundation	UK	Philanthropic
26.	Stroke Association	UK	Philanthropic
27.	The Epilepsy Fund (Epilepsiefonds)	Netherlands	Philanthropic
28.	US Department of Defense, Defense Health Agency & Congressionally Directed Medical Research Programs	USA	Public
29.	US Department of Health & Human Services, Agency for Healthcare Research and Quality	USA	Public
30.	Wellcome Trust	UK	Philanthropic
31.	ZonMw (Netherlands Organisation for Health Research and Development)	Netherlands	Public

Thirteen funders completed both the exploratory survey and the survey instrument. An additional file provides more details on respondents and non-respondents for both surveys (see Additional file 1).

#### Funders’ prioritization of dissemination and implementation

The majority of funder respondents (87%) reported that D&I of research findings is a high priority for their organizations. In an open-response question, funders were asked to explain why they agreed or disagreed that D&I is a high priority. Among the 87% of respondents that agreed that D&I is a high priority, 12 commented that D&I of research findings is crucial for maximizing the impact of funded research in terms of improved health care delivery and outcomes. Five respondents noted that, while they consider D&I to be a high priority, their organizations are still in the early stages of developing formal policies and structures to support D&I. For two, this process also includes consideration of internal resourcing.

Four respondents (13%) reported that D&I is not a high priority for their organizations. All four of these respondents were located in Europe, and two were public or government-funded organizations while the other two were philanthropic funders. Three were small funders and one was medium sized. In their open-response answer to explain why D&I is not a high priority for their organization, one respondent noted that they view D&I to be the responsibility of researchers rather than funders. The other three respondents indicated that they do not support active D&I strategies at this time but noted that their organizations do support release of findings, such as requiring awardees to publish research results.

#### Funders’ current level of activity supporting dissemination, implementation, and related practices

**Fig. 1:**
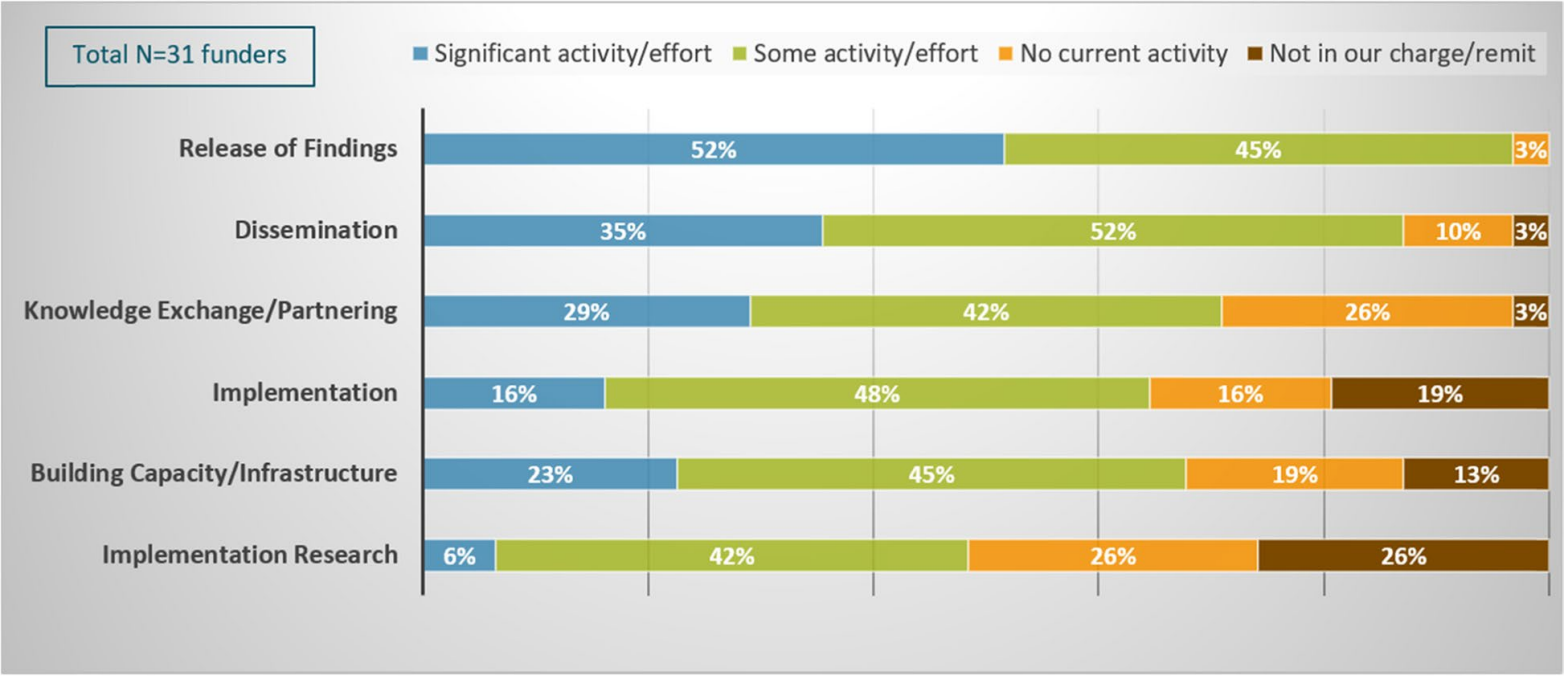
Funders’ level of activity/effort across six D&I practice areas. Respondents were asked to indicate the extent to which their organizations conduct activities within each of the six D&I practice areas shown on the vertical axis. Funders could select only one of the following four options: **a** “significant activity/effort,” **b** “some activity/effort,” **c** “no current activity,” or **d** the practice area is not in their organization’s “charge/remit.” The proportion of respondents that selected each option is shown for each D&I practice area

Over half of respondents reported that they have at least ‘some’ activity in the D&I practice areas surveyed, with the exception of implementation research. The vast majority of respondents indicated that they have “some” or “significant” activity in release of findings (97%) and dissemination (87%). In addition, most respondents reported that they have “some” or “significant” activity in knowledge exchange/partnering (71%), building capacity/infrastructure (68%), and implementation (64%). Funder respondents’ level of activity was lower for implementation research (48%); approximately one-fourth (26%) indicated that implementation research is outside their remit (Fig. 1).

#### Release of findings

While nearly all funder respondents (97%) reported at least “some” activity in the release of findings category, the specific types of activities supported varied. Sixteen respondents provide awardees with financial support to publish findings in academic journals, including support for open access fees, and five respondents also support publication deposit on open research platforms. For example, one respondent explained that they provide awardees with financial support for open access fees and that they fund a journals library to provide open access publication of complete findings from their major research programs.

In addition, 21 respondents reported that they share findings and information about their funded projects through their own channels, including their websites, social media accounts, newsletters, reports, and press briefings. To illustrate, one respondent noted that they share research results on social media and in their regular newsletter and that their website has a specific ‘impact stories’ section focusing on the outcomes of research. Nine respondents also support the translation of research findings into lay language for the general public. One respondent noted that they post all research findings to their webpage in the form of summaries for the lay public and for professionals. Overall, most respondents support release of findings through a combination of these activities.

#### Dissemination

A large majority of funders that responded to our survey (87%) indicated that they have “some” or “significant” activity/effort in the dissemination category, to actively spread research findings to target audiences. Funder respondents reported employing a variety of models to support dissemination. Thirteen respondents require or encourage research awardees to develop dissemination plans and conduct dissemination activities as part of the research project. Five respondents also support dissemination through separate funding schemes. For example, one respondent explained that they are considering the introduction of “follow-on dissemination awards” to enable enhanced and targeted dissemination activities at the end of a project funding period. In addition, 11 respondents develop targeted summaries, briefings, educational sessions, training materials, or other products to disseminate evidence to targeted audiences. For instance, one respondent described how they work closely with researchers to develop policy reports, briefings for members of parliament, and “lunch & learn” sessions that present new findings to service providers. Four respondents also noted that they work with developers of clinical practice guidelines or support the development of other tools to increase awareness of evidence among target audiences. Lastly, 15 respondents hold or provide financial support for conferences, workshops, webinars, etc. to disseminate research results to targeted audiences. As with the release of findings category, most respondents support dissemination through a combination of these different activities.

#### Knowledge exchange/partnering

Most funder respondents (71%) indicated that they have “some” or “significant” activity/effort in the knowledge exchange/partnering category, which involves convening stakeholders to share, respond to, and act upon research findings.^1^ Twelve respondents hold or support events to bring together researchers and knowledge users to discuss research results. For example, one respondent described their support of an exchange program to bring together researchers, implementation experts, and policymakers to discuss research evidence related to a health topic of shared interest. In addition, nine respondents stimulate partnerships between researchers and knowledge users by supporting specific knowledge exchange funding schemes and/or collaborations to help integrate research findings into policy and practice.

#### Implementation

Nearly two-thirds of funder respondents (64%) reported that they have “some” or “significant” activity in the implementation category, which involves support of efforts to integrate research findings within practice settings. Two respondents reported that they require or encourage research awardees to develop implementation plans, but did not mention any requirements for updates on actioning these. In addition, ten respondents provide funding for implementation activities, either as part of research awards or through separate funding schemes. Moreover, three respondents reported that they provide direct support to facilitate implementation of evidence-based interventions and services. For example, one respondent described how they are directly supporting the implementation of an intervention that was originally developed in a grant funded by the organization. Lastly, two respondents noted that although they currently have limited remits for implementation, they support integration of research findings indirectly through collaborations with stakeholders.

#### Building capacity/infrastructure

Most funder respondents (68%) indicated that they have “some” or “significant” activity in building capacity, to lay the groundwork for dissemination and implementation activities through investments in people and infrastructure. Nine respondents explained that they build capacity/infrastructure by providing funds to support career development and expertise in D&I. For instance, one respondent noted that they have fellowship awards to provide support to healthcare professionals, health systems personnel, health researchers, and health policy makers to develop the range of skills needed to translate evidence into healthcare and public health improvements. In addition, 12 respondents reported that they build capacity/infrastructure for D&I by investing in collaborations, networks, frameworks, tools, etc. to support D&I of research findings. For example, one respondent described how they provide funding to support the work of regional academic collaborative centers that bring together researchers, policymakers, teaching and practice institutions, and patients/clients to implement knowledge for use in policy and practice.

#### Implementation research

Compared to the other D&I practice areas, funder respondents reported the least amount of activity for implementation research. However, nearly half of respondents (48%) reported that they have “some” or “significant” activity/effort in this category. Four respondents noted that implementation research is within scope of their funding schemes; applicants may propose implementation research, but there is no specific call-out for it. Additionally, six respondents reported having dedicated funding for projects that assess the effectiveness or comparative effectiveness of D&I strategies. While implementation research appears to be the most limited D&I practice area in terms of activities and funding, several respondents expressed interest in developing implementation research programs and in collaborating with other funders to do so.

#### Funders’ characteristics related to extent of D&I activities

To examine potential trends in funders characteristics related to D&I activity we plotted the respondents’ size, region, and public or philanthropic character against the percentages of “some” or “significant” activity per category. The five large funders respondents report more activity than small funders respondents (*N*=16) in all the categories except Knowledge exchange/Partnering. Medium size funders respondents (*N*=9) also report more activity than small funders in the category Implementation. The four North American respondents all fund Implementation, Implementation research, and Knowledge Exchange/partnering. The three Australia/Pacific respondents all fund Building Capacity and Implementation. Respondents from Europe (other than the UK) and the UK report lower percentages in most categories. Public funders respondents (*n*=18) report to be more active than philanthropic funders respondents (*n*=13) in all categories (Table 3).

**Table 3:** Funder characteristics related to level of activity in four^a^ D&I categories (*N*=31)

Funder characteristics	Knowledge exchange/partnering %	Building capacity %	Implementation %	Implementation research %
**Funder size**^**b**^				
Small *n*=16	75	62	43	37
Medium *n*=9	88	66	88	44
Large *n*=5	80	80	80	80
(No budget indicated =1)				
**Region**				
Europe (other than UK) *n*= 16	88	50	56	44
UK *n*=8	63	88	50	38
North America *n*= 4	100	75	100	100
Australia/Pacific *n*=3	66	100	100	33
**Public/Philanthropic**				
Public *n*=18	83	72	72	61
Philanthropic *n*=13	77	62	54	31

The percentage of funders is shown per funder size, region, and public/philanthropic nature that report some or significant activity in a D&I category

^a^Categories release of findings and dissemination not included as almost all respondents engage in these activities

^b^Funder size based on reported annual budgets. Small: <€100 M, medium: €100M–€500M, large: > €500M. See Additional file 1: Appendix Table 3 for data per funder

#### Funders’ dissemination and implementation requirements for applicants

Funder respondents were asked to indicate whether their organizations require researchers to describe planned dissemination and/or implementation activities within their applications for research funding. The majority of respondents (81%) reported that they require all applicants to describe planned dissemination and/or implementation activities, and 16% indicated that they do so for certain funding schemes. Examples of specific requirements include questions in the funding applications regarding the end users that could benefit from the research and strategies for reaching these specific target audiences. Another example is requiring a description of the expected impact of the results through a pathway to impact plan.

#### Funders’ investment in dissemination and implementation

Funder respondents were asked whether their organizations have dedicated funds to support work in each area where they indicated activity. Among respondents that reported at least some activity, over half indicated that their organizations have dedicated funds for that area, with the exception of implementation research. The D&I area that had the highest proportion of respondents with dedicated funds was knowledge exchange/partnering (82%), followed by dissemination (70%), release of findings (67%), and building capacity/infrastructure (67%). Fewer respondents reported having dedicated funds for implementation (60%) and implementation research (40%).

In addition, the majority of respondents (90%) reported that their organizations specifically allot funding for D&I activities as part of research grants and/or upon successful completion of projects, mostly for dissemination activities and open access publication. Some organizations set a maximum amount or percentage of the project budget allowed for D&I activities, while others noted that their organizations do not set a predetermined amount for D&I activities.

Respondents were also asked to provide an estimate of the amount spent annually on D&I activities. Sixteen respondents (52%) indicated that they could not calculate an estimate, with several noting that their organizations do not track funds spent exclusively on D&I activities. For example, one respondent explained: “Many of these activities are integrated in the budget of research grants. Furthermore, our administration does not register these activities separately.” Among the 15 respondents that were able to provide an estimate, the amount spent annually on D&I activities ranged from <1% to 15% of the organization’s total annual budget, with an average of 5.2%.

Funder respondents were also asked if they had dedicated staffing for D&I activities. Forty-five percent of respondents (*n*=14) reported that their organizations have dedicated staff for D&I activities. Among respondents with dedicated D&I staff, the majority utilize staff with specialized skills in communications (93%), research (86%), public and patient involvement (64%), stakeholder engagement or advocacy (57%), and implementation science (57%). In addition, half of the respondents reported that their D&I staff have expertise in implementation/quality improvement practice, and 43% reported that their D&I staff have clinical expertise. The limited number of responding funders who were able to estimate the percentage of their annual budget (*n*=15) and staff (*n*=14) dedicated for D&I makes it difficult to determine trends relating funder size, public/private nature, or region to the investment in D&I activity.

#### Funders’ challenges related to conducting dissemination and implementation activities

**Fig. 2:**
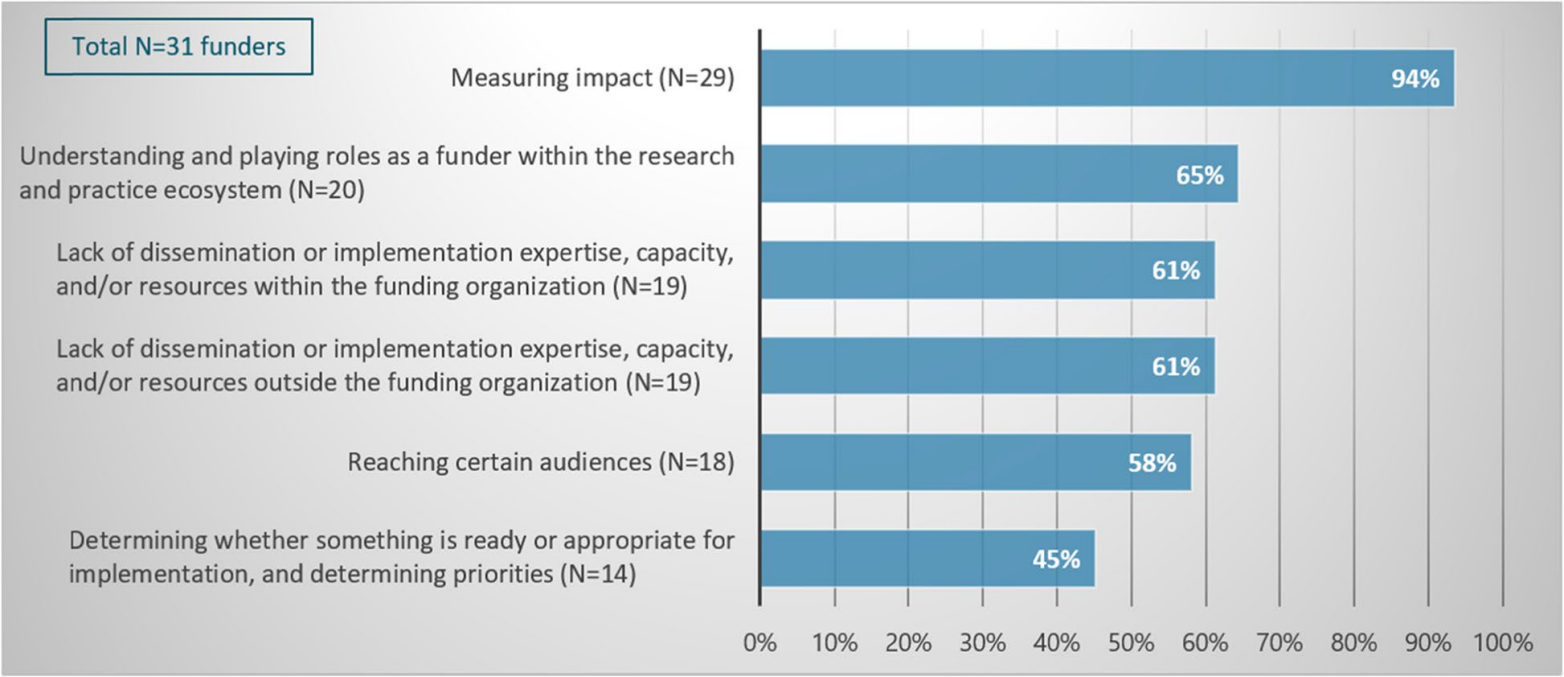
Challenges experienced by funders in conducting dissemination and implementation activities. Respondents were asked to indicate whether each of the issues listed on the vertical axis has been a challenge for their organization in terms of conducting D&I activities (“yes/no”). The graph shows the number and proportion of respondents that agreed that each D&I-related issue has been a challenge for their organization

Based on responses to the exploratory survey, the working group identified six types of D&I-related challenges that funders have encountered. In the survey instrument, funders were asked to indicate whether these issues have been a challenge for their organization and whether they experienced additional challenges. A large majority (94%) of respondents reported that ‘measuring impact’ has been a challenge for their organization. In addition, over half of respondents indicated that their organizations experience the following challenges: understanding the role of a funder in D&I within the research ecosystem (65%); lack of D&I expertise and/or resources both within the funding organization (61%) and outside the funding organization (61%); and reaching certain audiences (58%) (Fig. 2). Respondents named 16 additional challenges in the open-ended question. Most of these could be regrouped under the previously named categories but the challenge of visibility among researchers was named as a challenge not previously mentioned.

In addition, the majority of respondents noted that they would like to work collaboratively with other funders to address these challenges. Topics of interest to funders include how to reach different target audiences, effectiveness of D&I approaches, impact of different approaches, and expertise and/or infrastructure necessary for successful D&I.

## Discussion

The large majority of funders in this survey indicated that D&I is a priority for their organization. This high priority likely reflects a growing recognition of the importance of D&I activities in assuring that findings from research result in valuable improvements to health and well-being.

Despite this trend, our survey shows that fewer funders have a significant activity or dedicated funding for more active efforts to promote the uptake of findings, such as knowledge exchange and implementation, compared to more passive activities. Specifically, most funder respondents indicated a greater level of activity in the release of findings category and in targeted dissemination initiatives, while fewer funders indicated activities within the category of implementation. Further, despite the priority for D&I, fewer than half of funders surveyed indicated that they have dedicated staff for D&I. In addition, relatively few funders are undertaking implementation research that would support their own or others’ implementation efforts.

This survey’s results highlighted variation, reflecting different starting points, scope, and focus across funding organizations. These may shift and evolve over time. This limited descriptive research of selected funders provides a taxonomy of activity and baseline for future assessments and contributes to a growing literature on the role of funders and research intermediaries in getting research into practice.

Funders raised several challenges that they encounter while developing and carrying out D&I policies, from measuring impact to evaluating D&I activities, and respondents indicate a high level of willingness to collaborate on these within the EViR Funders’ Forum. The definitions and examples developed through this work may provide a foundation for the evaluation of various strategies used by funders in dissemination and implementation.

Since conducting the survey, an interest group has been created consisting of colleagues within EViR member organizations specifically responsible for conducting D&I policies. In 2021 the group has over 100 members from more than 20 different funding organizations and meets regularly to exchange their practices in the categories defined from this survey. This interest group aims to achieve further consensus on terminology, more insight into successful D&I practices, and may conduct collaborative evaluative research on D&I practices in the future.

The results of this survey were presented at the European Implementation Event (https://implementation.eu/european-implementation-event-2020/) in May 2021. Attendees at the meeting recommended that funders engage in regular interaction with research investigators on their views on the roles funders are taking in promoting D&I, as well as the support they need for conducting D&I.

## Limitations

This survey was distributed among funders linked to the EViR Funders’ Forum to provide insights into current activity and identify collaboration opportunities. Although EViR members include a range of funders—i.e., both public and philanthropic funders, range from small to large, and have limited to extensive remits for engaging in dissemination and implementation activities—the results of this survey cannot be regarded as representative of all funders internationally. EViR members are a self-selected group of funders, there may be selection bias. Not all countries are represented. The majority is from Europe and the UK; in particular, no funders from low-income countries are members of the Funders’ Forum. There are examples of large funders such as NIH in the US who are not EViR members and have not been surveyed, although they may conduct D&I activities. In addition, funders who are more active in D&I may have been more inclined to respond to this survey. While we report some differences in the extent of D&I activities related to size, region, and public/philanthropic nature with larger funders, North American and Australian/Pacific funders and public funders reporting more activities a wider survey including non EViR members would be necessary to determine if these trends are generalizable to all funders.

In addition, the limited number of responders who were able to estimate the percentage of their annual budget and have staff dedicated for D&I makes it difficult to determine trends relating funder characteristics to the investment in D&I activity based on the data from our responders’ group.

Although our iterative approach allowed for probing of areas of interest, the survey responses did not provide full detail regarding specific activities. Given the complex nature of D&I activities, some funders may not have fully articulated their activities.

## Conclusions

Although health research funders vary widely in mandate, funding source, and structure, all have an interest in assuring that funds invested in research ultimately help to improve health and well-being. This survey provides a useful baseline for funders in considering their priorities and activities in the context of what others are undertaking in the realm of dissemination and implementation. In addition, the findings from the survey and common definitions may allow for enhanced discussion among funders and other actors in the health research ecosystem, accelerating progress in dissemination and implementation more broadly.

## Supplementary Information

**Additional file 1.** Principle 10 Paper Appendix. 1. Exploratory Survey 2. Survey Instrument 3. Table All funders that received the exploratory survey and/or the survey instrument, and funder characteristics.

**Additional file 2.** Reporting guideline for survey research.

## Data Availability

Full agendas and minutes of the EViR Funders’ Forum meetings, redacted only to remove personal data, are either in the public domain on www.ensuringvalueinresearch.org or available on request. The datasets generated and/or analyzed during the current study are not publicly available due to the confidentiality responders were ensured regarding the non-publicly available data they provided but deidentified data are available from the corresponding author on reasonable request.

## Abbreviations

EViR: Ensuring Value in Research
D&I: Dissemination and implementation

## Footnotes

1 In the knowledge exchange/partnering category, several funders named examples of knowledge-user/stakeholder involvement in earlier stages of research, such as the research prioritization phase and during the execution of clinical studies. These funders did not provide details on how, if at all, this type of knowledge-user/stakeholder involvement contributes to D&I. Consequently, the working group determined that, while important and perhaps a prerequisite for successful D&I, these examples cannot be regarded as *part of* D&I and accordingly, did not include them in this report. The EViR Forum has a separate working group for knowledge-user/stakeholder involvement that is working on clarification of practices and collaboration opportunities in that area. The knowledge-user/stakeholder involvement examples named in the D&I survey have been forwarded to that working group.
