# Supplementary material for: Health funders’ dissemination and implementation practices: results from a survey of the Ensuring Value in Research (EViR) Funders’ Forum
Source: Implement Sci Commun. 2022 Mar 29;3:36. doi: 10.1186/s43058-022-00273-7 (PMC8966333; doi:10.1186/s43058-022-00273-7)
Supplement: Supplementary file 1 — Additional file 1. Principle 10 Paper Appendix. 1. Exploratory Survey 2. Survey Instrument 3. Table All funders that received the exploratory survey and/or the survey instrument, and funder characteristics. [file 43058_2022_273_MOESM1_ESM.docx]

Appendix

Table of Contents

[1. Exploratory Survey. 2](#_Toc84501593)

[2. Survey Instrument. 5](#_Toc84501594)

[3. Table All funders that received the exploratory survey and/or the survey instrument, and funder characteristics. 15](#_Toc84501595)

# Exploratory Survey.

**Ensuring Value in Research (EViR) Funders’ Collaboration and Development Forum Survey**

**Current practices, challenges, and opportunities for implementing**

**Principle 10:**

‘Research knowledge that can lead to benefit should be effectively disseminated to end users. Where appropriate, the usage of new knowledge should be supported and facilitated’.

As we have discussed at previous Forum meetings, the EViR guiding principles are intended to specify how we, as health-related research funders, can and should conduct organizational activities to increase the value of the research we fund. For each funder to achieve (or work towards achieving) these guiding principles different actions will be required, depending on their current practices, their research mandate, and the environment. For some principles, what we are doing already is sufficient; for some, it is clear what additional action we need to take; for others, we are uncertain and need examples of ways others are implementing this principle in order to improve our organizational approach. The examples developed by the Forum are intended to be illustrations of how a funder might adhere to the principles, within the limits of their capabilities or the nature of the research they fund, and is not an exhaustive list.

For furthering the work on Principle 10 a working group Dissemination and Implementation has been initiated. At a first meeting the working group expressed the wish to gain an overview on forum members practices regarding this principle. In addition, it was proposed to make an inventory of terminology and definitions used by Funders in the area of dissemination and or research usage as a wide variety of them can be found in literature and practice today.

This survey aims to gather information on your organization’s current practices related to Principle 10. **Please complete this survey and work with others in your organisation, as appropriate, to complete the questions below.** The working group will synthesize the information and share it at the next funders meeting and in follow-up emails to you.

We ask that you return the completed survey to Barbara van der Linden at [linden@zonmw.nl](mailto:linden@zonmw.nl) **no later than** **October 10^th^, 2018**. Thank you in advance for your participation.

| Question 1. Does your organisation have a remit for: |
| --- |
| Dissemination  Knowledge use  Implementation |
| Question 1a. Does the remit for the activities indicated above lie with the research funding team or with others in your organisation? If ‘others’, please explain.’ |

| Question 2. Which terms and/or definitions are in use in your organisation in the areas of dissemination and/or knowledge use/implementation? |
| --- |
|  |

| Question 3. What are your organisation’s current practices related to Principle 10? Does your organisation currently fund or support dissemination and/ or use of research findings? If so, please describe instruments, methods, activities and/or funding mechanisms by which you do this. If applicable please include links to websites, publications and/or other resources where your practices are described. |
| --- |
|  |

| Question 4. What are your organisation’s greatest challenges regarding implementation of Principle 10? Are there specific barriers that are the most vexing? Please describe. |
| --- |
|  |

| Question 5. Are there specific areas of Principle 10 where working with other funders could enhance or improve your organisation’s practice? Are there specific best practices or models you have been seeking? Please describe. |
| --- |
|  |

Thank you for taking the time to fill in this survey, please send it to Barbara van der Linden at [linden@zonmw.nl](mailto:linden@zonmw.nl) before **September 30^th^ 2018.**

# Survey Instrument.

**Ensuring Value in Research (EViR) Funders’ Collaboration and Development Forum: Current practices, challenges, and opportunities for implementation of Principle 10 (P10)**

***Principle 10 Survey, Round 2***

As we have discussed at previous Forum meetings, the EViR Guiding Principles are intended to specify how we, as health-related research funders, can and should conduct organizational activities to increase the value of the research we fund. To better understand practices and foster collaboration, we occasionally distribute surveys to characterize current practices among funders.

EViR members have rated dissemination and implementation as high-priority activities. In August 2018, we distributed the first round of the survey on Guiding Principle 10 (P10). The survey yielded a broad view of what funders regard as dissemination and implementation and the types of practices they carry out.

With this second round of the survey, we seek a more systematic overview of funders’ practices and the challenges they experience in conducting dissemination and implementation activities. This information will be used to identify areas for collaboration and to better understand how to improve the reach, uptake, and relevance of research outputs for a range of audiences. In order to facilitate exchange and learning, we are looking for insightful descriptions of your practices in these areas.

We ask that you work with colleagues in your organization, as appropriate, to complete this survey. Each organization should submit only one response. **Please complete the survey no later than July 29, 2019.** We estimate that the survey will take 30-60 minutes to complete, depending on the amount of detail you supply**.**

The P10 Working Group will synthesize the survey results and share findings at the **next EViR meeting in Washington, DC on September 19-20, 2019**. Wider sharing of the results will be reported in aggregate form only; if we are interested in sharing individual examples, then we will reach out for your consent.

If you have any questions, please contact [Emily Lazowick](mailto:elazowick@pcori.org) at PCORI. Thank you in advance for your participation!

| **Principle 10** | **Examples of Implementation** |
| --- | --- |
| *Research knowledge that can lead to benefit should be effectively disseminated to end users. Where appropriate, the usage of new knowledge should be supported and facilitated****.*** | - Funders could financially or materially support the dissemination of research findings. - Funders could ensure that a summary of the study conduct, analysis and findings are provided in a format accessible to a lay audience. - Funding for implementation research or the implementation of new findings into practice could be available. |

1. **What is the name of your organization?** Click or tap here to enter text.
2. **Who is the contact person for this survey?** Click or tap here to enter text.
3. **Please provide the contact person’s email address.** Click or tap here to enter text.

***Please continue onto the next page***

1. **Please indicate the extent to which your organization conducts activities within each of the P10 practice areas listed below, and briefly describe your current activities in each P10 area. *Please refer to the table that was sent as a separate email attachment for examples of activities in each P10 area.***

| **P10 Practice Areas** | **Not in our charge/remit** | **No current activity** *(future activity may be planned)* | **Some activity/effort** *(limited in scope or investment)* | **Significant activity/effort** | **Describe your current activities** (no more than 4-5 sentences. The final survey question will allow for more detailed descriptions of your practices). |
| --- | --- | --- | --- | --- | --- |
| **Release of Findings:**  Diffusion, passive activities, and supportive policies to make research findings available and accessible to the general public and other audiences | 1 | 2 | 3 | 4 |  |
| **Dissemination:**  An active approach of spreading research findings to the target audience via determined channels using planned strategies | 1 | 2 | 3 | 4 |  |
| **Knowledge Exchange/Partnering:**  Actively bringing stakeholders together  to share, respond to, and act upon research  findings | 1 | 2 | 3 | 4 |  |
| **Implementation:**  Active and planned efforts to use or integrate research findings within a setting | 1 | 2 | 3 | 4 |  |
| **Building Capacity/Infrastructure for Dissemination or Implementation:**  Investing in people and structures that enable/lay the groundwork for dissemination and/or implementation of research evidence | 1 | 2 | 3 | 4 |  |
| **Implementation Research:**  Investment in and/or carrying out research on determinants/models/working methods and effectiveness of dissemination & implementation efforts or initiatives | 1 | 2 | 3 | 4 |  |

1. **Would you say that dissemination and implementation of research findings is a high priority for your organization?**
   1. Yes
   2. No
2. **Please explain your answer to the question above.**

[open response] Click or tap here to enter text.

***Please continue onto the next page***

1. **Please indicate whether your organization has dedicated funds for activities in each of the P10 practice areas listed below. *Please refer to the table that was sent as a separate email attachment for examples of activities in each P10 area.***

| **P10 Practice Areas** | *Does your organization have dedicated funds for activities in this P10 area?* | |
| --- | --- | --- |
|  | **Yes** | **No** |
| **Release of Findings:**  Diffusion, passive activities, and supportive policies to make research findings available and accessible to the general public and other audiences | 1 | 2 |
| **Dissemination:**  An active approach of spreading research findings to the target audience via determined channels using planned strategies | 1 | 2 |
| **Knowledge Exchange/Partnering:**  Actively bringing stakeholders together  to share, respond to, and act upon research findings | 1 | 2 |
| **Implementation:**  Active and planned efforts to use or integrate research findings within a setting | 1 | 2 |
| **Building Capacity/Infrastructure for Dissemination or Implementation:**  Investing in people and structures that enable/lay the groundwork for dissemination and/or implementation of research evidence | 1 | 2 |
| **Implementation Research:**  Investment in and/or carrying out research on determinants/models/working methods and effectiveness of dissemination & implementation efforts or initiatives | 1 | 2 |

1. **Approximately how much money does your organization spend annually on P10-related activities (dissemination, implementation, etc.) (best estimate)? Please explain how you derived this estimate.**

[open response] Click or tap here to enter text.

1. **What is your organization’s overall annual budget?**

[open response] Click or tap here to enter text.

1. **Does your organization have dedicated staff for dissemination & implementation activities?**
   1. Yes
   2. No

**Please answer Questions 11 and 12 *only* if you answered “Yes” to Question 10 above.**

1. **Approximately what percentage of total FTEs does your organization have dedicated for dissemination & implementation activities? Please explain how you arrived at your answer.**

[open response] Click or tap here to enter text.

1. **Please indicate the skill sets or expertise of those who work on dissemination & implementation activities for your organization. Please select all that apply.**

- Communications
- Clinical
- Research
- Implementation Science
- Implementation/Quality Improvement Practice
- Public and Patient Involvement
- Stakeholder Engagement or Advocacy
- Other – Write In Click or tap here to enter text.

1. **Do you require researchers to describe planned dissemination and/or implementation activities within their applications for funding? If your answer is “yes” or “in some cases,” please describe your requirements in a few sentences.**
   1. Yes Click or tap here to enter text.
   2. In some cases Click or tap here to enter text.
   3. No
2. **Does your organization fund dissemination and implementation activities as part of research grants and/or upon successful completion of research projects? If yes, please describe in a few sentences (e.g. % of budget allowance, limited or open competition, etc.).**
   1. Yes, *only* as part of research grants Click or tap here to enter text.
   2. Yes, *only* upon completion of research project Click or tap here to enter text.
   3. Yes, as part of research grants *and/or* upon completion of research project Click or tap here to enter text.
   4. No
3. **CHALLENGES: Thinking about the experience of your organization, please indicate the extent to which you agree or disagree that the following issues have been a challenge to implementation of Principle 10.**

|  | *Indicate the extent to which you agree or disagree that the following issues have been a challenge for your organization. Please select one option.* | | | *Are you interested in working with other funders on this challenge?* | |
| --- | --- | --- | --- | --- | --- |
| **Issues** | **Not applicable** | **Challenge for our organization** | **Not a challenge for our organization** | **Yes** | **No** |
| Lack of dissemination or implementation expertise, capacity, and/or resources *within* the funding organization | 1 | 2 | 3 | 1 | 2 |
| Lack of dissemination or implementation expertise, capacity, and/or resources *outside* the funding organization | 1 | 2 | 3 | 1 | 2 |
| Determining whether something is ready or appropriate for implementation, and determining priorities | 1 | 2 | 3 | 1 | 2 |
| Reaching certain audiences | 1 | 2 | 3 | 1 | 2 |
| Understanding and playing roles as a funder within the research and practice ecosystem | 1 | 2 | 3 | 1 | 2 |
| Measuring impact | 1 | 2 | 3 | 1 | 2 |

1. **Are there any other issues that have been a challenge for conducting dissemination & implementation activities at your organization? If so, please explain.**

[open response] Click or tap here to enter text.

1. **Research on Key Uncertainties**

Would research on key uncertainties related to dissemination & implementation improve your organization’s practice? The research needs listed below have been identified by EViR participants in the past. **Please indicate your interest in research on any of the following topics (select all that apply). You can also add additional topics in the “Other” textbox.**

- Reaching different target audiences
- Effectiveness of Dissemination & Implementation approaches
- Impact of Dissemination & Implementation approaches
- Expertise and/or infrastructure necessary for successful Dissemination & Implementation
- Other – Write In Click or tap here to enter text.

1. **Please describe one or two good examples of your organization’s practices within the area of dissemination or implementation. *Please limit your response to approximately 200 words per textbox.***

|  | **Good Practice Example 1** | **Good Practice Example 2** |
| --- | --- | --- |
| Name of Practice |  |  |
| Purpose of Practice |  |  |
| Description of Practice  (please include target audiences) |  |  |
| Resources (budget, staff, etc.) your organization uses for this practice |  |  |
| Resources your organization needs to sustain or enhance this practice |  |  |
| Effectiveness/Impact of Practice |  |  |
| Lessons Learned/Reflections |  |  |
| Links for additional information |  |  |

***THANK YOU FOR YOUR PARTICIPATION***

# Table All funders that received the exploratory survey and/or the survey instrument, and funder characteristics.

|  | **Funder** | **Country** | **Region** | **Received Exploratory Survey?** | **Responded to Exploratory Survey?** | **Received Survey Instrument?** | **Responded to Survey Instrument?** | **Public vs. Philanthropic** | **Condition-specific vs. Non-specific** | **Funder Size Based on Overall Annual Budget (self-reported):**  **Small: 0-100M€**  **Medium: 100-500M€**  **Large: >500M€** |
| --- | --- | --- | --- | --- | --- | --- | --- | --- | --- | --- |
|  | Aidsfonds | Netherlands | Europe | No | N/A | Yes | Yes | Philanthropic | Specific | Small |
|  | Alzheimer Nederland | Netherlands | Europe | No | N/A | Yes | Yes | Philanthropic | Specific | Small |
|  | Arthritis Research UK | United Kingdom | Europe | Yes | No | No | N/A | Philanthropic | Specific | N/A |
|  | Australian Government Department of Health, Office of Health and Medical Research | Australia | Australia Pacific | No | N/A | Yes | Yes | Public | Non-specific | Medium |
|  | Austrian Science Fund (FWF) | Austria | Europe | Yes | No | Yes | No | Public | Non-specific | N/A |
|  | Belgian Health Care Knowledge Centre (KCE) | Belgium | Europe | No | N/A | Yes | No | Public | Non-specific | N/A |
|  | Brain Foundation Netherlands (Hersenstichting) | Netherlands | Europe | No | N/A | Yes | Yes | Philanthropic | Specific | Small |
|  | British Heart Foundation | United Kingdom | Europe | No | N/A | Yes | No | Philanthropic | Specific | N/A |
|  | Bupa Health Foundation | Australia | Australia Pacific | No | N/A | Yes | No | Philanthropic | Non-specific | N/A |
|  | Canadian Institutes of Health Research | Canada | North America | Yes | Yes | Yes | Yes | Public | Non-specific | Large |
|  | Cancer Research UK | United Kingdom | Europe | No | N/A | Yes | No | Philanthropic | Specific | N/A |
|  | Commonwealth Scientific and Industrial Research Organisation (CSIRO) | Australia | Australia Pacific | No | N/A | Yes | No | Public | Non-specific | N/A |
|  | Diabetes Fonds | Netherlands | Europe | No | N/A | Yes | Yes | Philanthropic | Specific | Small |
|  | Dutch Arthritis Society (ReumaNederland) | Netherlands | Europe | No | N/A | Yes | No | Philanthropic | Specific | N/A |
|  | Dutch Burns Foundation (Brandwondenstichting) | Netherlands | Europe | No | N/A | Yes | No | Philanthropic | Specific | N/A |
|  | Dutch Cancer Society (Kankerbestrijding) | Netherlands | Europe | No | N/A | Yes | No | Philanthropic | Specific | N/A |
|  | Dutch Cystic Fibrosis Foundation (NCFS) | Netherlands | Europe | No | N/A | Yes | No | Philanthropic | Specific | N/A |
|  | Dutch Heart Foundation (Hartstichting) | Netherlands | Europe | Yes | Yes | Yes | Yes | Philanthropic | Specific | Small |
|  | Dutch Kidney Foundation (Nierstichting Nederland) | Netherlands | Europe | No | N/A | Yes | Yes | Philanthropic | Specific | Small |
|  | Dutch MS Research Foundation (Stichting MS Research) | Netherlands | Europe | No | N/A | Yes | No | Philanthropic | Specific | N/A |
|  | Forte: Swedish Research Council for Health, Working Life and Welfare | Sweden | Europe | Yes | Yes | Yes | Yes | Public | Non-specific | Small |
|  | German Federal Ministry of Education and Research, DLR Project Management Agency | Germany | Europe | Yes | Yes | Yes | Yes | Public | Non-specific | Medium |
|  | German Research Foundation (Deutsche Forschungsgemeinschaft) | Germany | Europe | Yes | Yes | Yes | No | Public | Non-specific | N/A |
|  | Health and Care Research Wales | United Kingdom | Europe | Yes | Yes | Yes | Yes | Public | Non-specific | Small |
|  | Health and Social Care in Northern Ireland, Public Health Agency | United Kingdom | Europe | Yes | Yes | Yes | Yes | Public | Non-specific | Small |
|  | Health Research Board Ireland | Ireland | Europe | Yes | Yes | Yes | Yes | Public | Non-specific | Small |
|  | Health Research Council of New Zealand | New Zealand | Australia Pacific | Yes | Yes | Yes | Yes | Public | Non-specific | Small |
|  | Longfonds (The Lung Foundation Netherlands) | Netherlands | Europe | No | N/A | Yes | No | Philanthropic | Specific | N/A |
|  | Marie Curie | United Kingdom | Europe | Yes | Yes | Yes | Yes | Philanthropic | Specific | Medium |
|  | Medical Research Council | United Kingdom | Europe | Yes | No | Yes | Yes | Public | Non-specific | Large |
|  | Melanoma Research Alliance | US | North America | Yes | No | Yes | No | Philanthropic | Specific | N/A |
|  | Mental Health Foundation | United Kingdom | Europe | Yes | No | Yes | No | Philanthropic | Specific | N/A |
|  | MIND | Netherlands | Europe | No | N/A | Yes | Yes | Philanthropic | Specific | Small |
|  | Ministry of Health, Italy | Italy | Europe | Yes | No | Yes | Yes | Public | Non-specific | Medium |
|  | National Health and Medical Research Council | Australia | Australia Pacific | Yes | No | Yes | Yes | Public | Non-specific | N/A |
|  | National Institute for Health Research | United Kingdom | Europe | Yes | Yes | Yes | Yes | Public | Non-specific | Large |
|  | National Institutes of Health | United States | North America | Yes | No | Yes | No | Public | Non-specific | N/A |
|  | National Science Centre | Poland | Europe | Yes | No | Yes | Yes | Public | Non-specific | Medium |
|  | Netherlands Thrombosis Foundation (Trombosestichting) | Netherlands | Europe | No | N/A | Yes | No | Philanthropic | Specific | N/A |
|  | NHS Scotland, Chief Scientist Office | United Kingdom | Europe | Yes | No | Yes | No | Public | Non-specific | N/A |
|  | PCORI | United States | North America | Yes | Yes | Yes | Yes | Public | Non-specific | Medium |
|  | Princess Beatrix Muscle Fund (Prinses Beatrix Spierfonds) | Netherlands | Europe | No | N/A | Yes | Yes | Philanthropic | Specific | Small |
|  | Research Council of Norway | Norway | Europe | Yes | No | Yes | Yes | Public | Non-specific | Medium |
|  | Scar Free Foundation | United Kingdom | Europe | Yes | No | Yes | Yes | Philanthropic | Specific | Small |
|  | Stomach Liver Bowel Foundation (Maag Lever Darm Stichting) | Netherlands | Europe | No | N/A | Yes | No | Philanthropic | Specific | N/A |
|  | Stroke Association | United Kingdom | Europe | Yes | Yes | Yes | Yes | Philanthropic | Specific | Small |
|  | Swiss National Science Foundation | Switzerland | Europe | Yes | No | Yes | No | Public | Non-specific | N/A |
|  | The Epilepsy Fund (Epilepsiefonds) | Netherlands | Europe | No | N/A | Yes | Yes | Philanthropic | Specific | Small |
|  | The Québec Research Funds | Canada | North America | Yes | No | Yes | No | Public | Non-specific | N/A |
|  | US Department of Defense, Defense Health Agency and Congressionally Directed Medical Research Programs | United States | North America | Yes | No | Yes | Yes | Public | Non-specific | Large |
|  | US Department of Health and Human Services, Agency for Healthcare Research and Quality | United States | North America | Yes | No | Yes | Yes | Public | Non-specific | Medium |
|  | US Department of Veterans Affairs, Office of Research and Development | United States | North America | Yes | No | Yes | No | Public | Non-specific | N/A |
|  | Wellcome Trust | United Kingdom | Europe | Yes | No | Yes | Yes | Philanthropic | Non-specific | Large |
|  | ZonMw (Netherlands Organisation for Health Research and Development) | Netherlands | Europe | Yes | Yes | Yes | Yes | Public | Non-specific | Medium |

N/A: not available
