# Supplementary material for: Health funders’ dissemination and implementation practices: results from a survey of the Ensuring Value in Research (EViR) Funders’ Forum
Source: Implement Sci Commun. 2022 Mar 29;3:36. doi: 10.1186/s43058-022-00273-7 (PMC8966333; doi:10.1186/s43058-022-00273-7)
Supplement: Supplementary file 2 — Additional file 2. Reporting guideline for survey research. [file 43058_2022_273_MOESM2_ESM.docx]

Additional file 2: Reporting guideline for survey research

This reporting checklist was extracted from the following article found on the EQUATOR Network:

Kelley K, Clark B, Brown V, Sitzia J. Good practice in the conduct and reporting of survey research. Int J Qual Health Care. 2003;15(3):261-6.

<https://www.equator-network.org/reporting-guidelines/good-practice-in-the-conduct-and-reporting-of-survey-research/>

When reporting survey research, it is essential that a number of key points are covered (though the length and depth of reporting will be dependent upon journal style). These key points are presented as a ‘checklist’ below:

1. Explain the purpose or aim of the research, with the explicit identification of the research question**. Described in Background section, pages 3-4**
2. Explain why the research was necessary and place the study in context, drawing upon previous work in relevant fields (the literature review). **Described in Background section, pages 3-4**
3. Describe in (proportionate) detail how the research was done.  **All of the following items, aside from one marked as N/A, are described in the Methods section, pages 4-7**
   - - 1. State the chosen research method or methods, and justify why this method was chosen.
       2. Describe the research tool. If an existing tool is used, briefly state its psychometric properties and provide references to the original development work. If a new tool is used, you should include an entire section describing the steps undertaken to develop and test the tool, including results of psychometric testing.
       3. Describe how the sample was selected and how data were collected, including:
     1. How were potential subjects identified?
     2. How many and what type of attempts were made to contact subjects?
     3. Who approached potential subjects?
     4. Where were potential subjects approached?
     5. How was informed consent obtained? **N/A (study was determined to be exempt from oversight by the Advarra institutional review board).**
     6. How many agreed to participate?
     7. How did those who agreed differ from those who did not agree?
     8. What was the response rate?
4. Describe and justify the methods and tests used for data analysis. **Described in Methods section, pages 4-7**
5. Present the results of the research. The results section should be clear, factual, and concise. **Described in Results section, pages 7-15**
6. Interpret and discuss the findings. This ‘discussion’ section should not simply reiterate results; it should provide the author’s critical reflection upon both the results and the processes of data collection. The discussion should assess how well the study met the research question, should describe the problems encountered in the research, and should honestly judge the limitations of the work. **Described in Discussion section, pages 16-17**
7. Present conclusions and recommendations. **Described in Conclusions section, page 17**
